# Bioactive Glass and Melittin Thin Films Deposited by MAPLE for Titanium Implant Functionalization

**DOI:** 10.3390/ma18102410

**Published:** 2025-05-21

**Authors:** Mihaela Dinu, Bogdan Bita, Anca Constantina Parau, Carmen Ristoscu, Irina Negut

**Affiliations:** 1 National Institute of Research and Development for Optoelectronics—INOE2000, 409 Atomistilor St., 077125 Magurele, Romania; mihaela.dinu@inoe.ro (M.D.); bogdan.bita@inoe.ro (B.B.); anca.parau@inoe.ro (A.C.P.); 2 Faculty of Physics, University of Bucharest, 405 Atomistilor, 077125 Magurele, Romania; 3 National Institute for Laser, Plasma and Radiation Physics, 409 Atomistilor Street, P.O. Box MG 36, 077125 Magurele, Romania; carmen.ristoscu@inflpr.ro

**Keywords:** bioglass, melittin, matrix-assisted pulsed laser evaporation, titanium/orthopedic implants, bone regeneration

## Abstract

The development of bioactive coatings for metallic implants is essential to enhance osseointegration and improve implant longevity. In this study, composite thin films based on bioactive glass and melittin were synthesized using the matrix-assisted pulsed laser evaporation technique and deposited onto titanium substrates. The coatings were characterized using physicochemical analysis methods, including scanning electron microscopy, atomic force microscopy, contact angle measurements, Fourier transform infrared spectroscopy, energy-dispersive X-ray spectroscopy, and electrochemical impedance spectroscopy. Simulated body fluid immersion tests were also conducted to assess bioactivity over time. Scanning electron microscopy and atomic force microscopy revealed dense, irregular surface textures with nanoscale features and an average roughness of ~120 nm, favorable for cell adhesion. Contact angle measurements showed a significant shift from hydrophobic (~95° for bare titanium) to moderately hydrophilic (~62° for the bioglass and melittin coating) surfaces, indicating improved biocompatibility. Electrochemical impedance spectroscopy demonstrated enhanced corrosion resistance in simulated body fluid, with the coating exhibiting a ~45% decrease in impedance magnitude after 12 h of immersion, compared to only 4% for bare titanium. Fourier transform infrared spectroscopy and energy-dispersive X-ray spectroscopy analyses confirmed the progressive formation of a carbonated apatite layer after 7 days of simulated body fluid exposure, suggesting high bioactivity and osteoconductive potential. The combined effects of bioactive glass and melittin in the thin film structure offer promising applications in orthopedic and dental implants, enhancing both biological performance and structural integrity.

## 1. Introduction

Achieving rapid and long-lasting osseointegration is a critical requirement for the success of orthopedic and dental implants. Osseointegration refers to the direct structural and functional connection between living bone and the surface of an implant, and it is essential for the implant’s mechanical stability and longevity. Despite advances in biomaterials, titanium (Ti) and its alloys, which are widely used for implants due to their excellent mechanical properties and corrosion resistance, are biologically inert. This lack of bioactivity often delays or limits bone–implant bonding, leading to risks of implant failure, loosening, or the need for revision surgery [1,2,3].

In recent decades, the integration of bioactive materials with metallic substrates has gained growing importance in biomedical engineering [4], particularly in the development of coatings for orthopedic and dental implants.

Surface modification with bioactive coatings can enhance cell adhesion, proliferation, and differentiation at the bone–implant interface, thereby improving clinical outcomes.

Surface modifications such as hydrothermal treatment have been shown to create nanoporous architectures that significantly enhance osteogenic differentiation of stem cells by modulating surface wettability and nanoscale morphology [5]. Additionally, Mg-containing coatings have gained attention for promoting bone regeneration through activation of key signaling pathways, thus enhancing osteoblast activity [6]. Recent reviews also emphasize the effectiveness of nanohydroxyapatite coatings, which mimic natural bone mineral and have demonstrated significant improvements in osteoblast attachment and bone integration [7].

Beyond physical surface modifications, biomolecular coatings, such as collagen-mimetic peptides and integrin-targeted ligands, have been employed to directly stimulate osteogenic differentiation from progenitor cells at the implantation site [8]. Furthermore, dual-functionalized coatings combining cell-adhesive and osteogenic peptides have shown promising results in promoting osteoblast proliferation and bone matrix formation even under conditions such as osteoporosis [9]. These approaches show the importance of not only enhancing the physical and chemical characteristics of implant surfaces but also biochemically guiding stem cell differentiation to achieve optimal osseointegration.

Bioactive glasses (BGs) stand out due to their exceptional biocompatibility, osteoconductivity, and ability to form a direct bond with bone tissue through the formation of a hydroxyapatite-like layer in physiological conditions [10]. Despite their promising bioactivity, bulk BGs are inherently brittle and mechanically weak, which limits their direct application as load-bearing materials. To overcome this limitation, thin-film deposition of BGs onto mechanically robust substrates such as Ti has emerged as a compelling strategy.

BG coatings have been widely explored for their ability to enhance osseointegration and promote bone tissue regeneration [10]. For example, Costa et al. introduced a novel plasma electrolytic oxidation method to develop BG-based coatings on Ti, which demonstrated superior corrosion resistance, high protein adsorption, and excellent biocompatibility, which represent key factors for successful implant integration [11]. Liang et al. systematically reviewed various coating technologies, including pulsed laser deposition and electrophoretic deposition, highlighting the significant improvements in osseointegration, osteogenesis, and angiogenesis achieved through BG coatings [10]. AlMaimouni et al. emphasized that BG-coated dental implants rapidly form apatite layers, enhancing cell attachment and proliferation while also providing corrosion protection [12]. Recent research shows that BG coatings not only promote apatite formation but also improve the corrosion resistance of Ti implants [13,14]. Advanced BG compositions and multilayer coatings have been shown to enhance osteoconductivity while mitigating issues related to stress shielding [10].

Complementing the osteogenic potential of BGs, Zarghami et al. [15,16] developed composite coatings incorporating Mel and demonstrated an enhancement in osteoblast-like cell proliferation and differentiation, offering a multifunctional solution for both infection prevention and bone regeneration [15,16]. Mohammed also reported that Ti implants coated with pulsed laser-deposited BG exhibited enhanced bioactivity and biocompatibility, showing the clinical potential of such hybrid coatings [17].

One advanced method for depositing delicate organic-inorganic composite coatings is matrix-assisted pulsed laser evaporation (MAPLE). This technique enables the gentle transfer of thermally sensitive biomolecules without degrading their structure, making it ideal for functionalizing implants with bioactive peptides or polymers [18].

The current study builds upon this principle by incorporating melittin (Mel), a biologically active peptide known for its antimicrobial properties and cell membrane interactions, into a BG-based coating. Mel’s amphiphilic nature allows it to modulate cell behavior and contribute to surface hydrophilicity—both crucial for enhancing biocompatibility and early-stage osseointegration [19,20].

Incorporating BG57, a silicate-based BG, into the thin film structure provides a rich source of calcium and phosphate ions, essential for initiating the mineralization process on implant surfaces. Upon immersion in simulated body fluid (SBF), BG57 undergoes controlled dissolution, releasing ions that induce the nucleation and growth of an apatite layer. This mineral layer is structurally and compositionally similar to natural bone, thereby facilitating stronger bonding between the implant and host tissue [21].

In addition to structural integration, implant coatings must resist electrochemical degradation over time. Corrosion resistance is particularly important in physiological environments rich in chloride ions, which can penetrate micro-defects and compromise the longevity of the implant [22,23]. By enhancing surface wettability and forming protective mineral layers, BG+Mel coatings aim to reduce susceptibility to corrosion and prolong functional implant life.

Given the promising individual characteristics of BG57 and Mel, their combination into a single coating system via MAPLE represents a novel approach to engineering multifunctional implant surfaces. This study aims to evaluate the physicochemical, morphological, and electrochemical behavior of BG57+Mel films deposited on Ti, with particular focus on their bioactivity in SBF and potential for future biomedical applications in bone regeneration. Based on the objectives of this work, the following null hypotheses were formulated: (i) the BG57+Mel coating does not significantly alter the surface morphology, wettability, or electrochemical behavior of Ti; and (ii) the BG57+Mel coating does not enhance in vitro bioactivity as evidenced by apatite formation in SBF. These hypotheses are tested and refuted through the results presented in this study.

## 2. Materials and Methods

### 2.1. Materials

All analytical-grade reagents—including acetone (C_6_H_6_O), ethanol (C_2_H_5_OH), dimethyl sulfoxide (DMSO), and the full set of chemicals required for simulated body fluid (SBF) synthesis, such as NaCl, NaHCO_3_, KCl, K_2_HPO_4_·3H_2_O, MgCl_2_·6H_2_O, HCl, CaCl_2_, Na_2_SO_4_, and tris(hydroxymethyl)aminomethane, were procured from Sigma-Aldrich GmbH (Steinheim, Germany). Mel was also acquired from Sigma-Aldrich GmbH (Steinheim, Germany). The SBF solution, designed to closely mimic the ionic composition of human blood plasma, was prepared following the modified protocol developed by Kokubo [24], ensuring precise sequencing and quantities of each reagent during preparation.

The BG57 powders were prepared following the methods outlined in references [25,26]. These powders belong to the compositional system comprising SiO_2_–Na_2_O–K_2_O–CaO–MgO–P_2_O_5_.

Grade 4 titanium (Ti) foils, measuring (0.8 × 0.8) cm^2^, were employed as substrates for thin film deposition. Prior to use, they underwent a standardized cleaning procedure involving sequential 15 min ultrasonic baths in acetone, ethanol, and deionized water (Elma Schmidbauer GmbH, Singen, Germany). The substrates were then dried using high-purity nitrogen gas (N_2_, 5.0 grade). After drying, they were carefully affixed onto sample holders and placed into the deposition chamber. Ti was chosen due to its excellent biocompatibility and established use in bone-related medical implants [27,28,29].

### 2.2. Methods

The organic composite BG57 combined with Mel was prepared as a target material by dissolving 0.06 g of BG57 and 60 µg of Mel in 10 mL of DMSO. Zarghami et al. demonstrated that Mel coatings containing 0.05 µg/mL in combination with antibiotics effectively eradicated MRSA and VRSA biofilms while reducing cytotoxicity toward osteoblast cells [15]. Similarly, Rai et al. developed antimicrobial coatings using immobilized melittin derivatives at surface densities of <10–110 µg/cm^2^, achieving broad antibacterial activity with low cytotoxicity against mammalian cells [30]. Additionally, Maher and McClean et al. reported that Mel at submicromolar concentrations (~0.3 µg/mL) enhanced cell proliferation, whereas higher doses triggered necrotic cytotoxicity in epithelial cells [31]. Similarly, Wu et al. observed that native Mel was cytotoxic to mammalian Caco-2 cells above 2 µg/mL, highlighting a narrow therapeutic window [32]. Previous studies have shown that Mel exhibits cytotoxicity toward mammalian cells (IC_50_ values as low as 2–6 µg/mL for various cell lines) and significant hemolytic activity even at concentrations below 1 µg/mL [19]. Higher doses of Mel have also been linked to calcium influx-induced apoptosis in osteoblast-like cells [33]. Moreover, Mel’s minimum inhibitory concentration (MIC) against pathogens such as *S. aureus* and *E. coli* ranges from 0.3–32 µg/mL, depending on the strain [32], but achieving these concentrations locally without triggering unwanted toxicity in adjacent mammalian tissues remains a major challenge. Therefore, we selected a low Mel loading to ensure structural incorporation in the thin film while minimizing its uncontrolled release and reducing the risk of cytotoxicity in vivo. Additionally, a prior study on immobilized antimicrobial peptide coatings reported antimicrobial efficacy at surface Mel densities as low as 1–10 µg/cm^2^ without compromising mammalian cell viability [30]. Our Mel content aligns with this range when considering the thin film’s nanoscale thickness and surface coverage.

Considering these findings, we selected a low Mel loading (~60 µg) to achieve antimicrobial potential at the implant surface while avoiding excessive release that could lead to cytotoxicity or hemolysis in adjacent tissues. The thin film architecture and immobilization within the BG matrix further limit melittin’s systemic diffusion, consistent with strategies reported in nanoparticle and immobilized peptide systems to reduce Mel’s inherent toxicity [34].

DMSO solvent was selected for its high vapor pressure and strong absorption in the ultraviolet range. The resulting mixture was cast onto a copper substrate and rapidly frozen in a liquid nitrogen bath to form solid targets. These frozen targets were then mounted onto a cooled holder inside the vacuum deposition system, where they were maintained at liquid nitrogen temperature throughout the deposition process. To promote uniform film growth and prevent localized damage, the cryogenic target was rotated at a constant rate of 50 rpm during laser exposure.

While ultraviolet laser irradiation often leads to thermal degradation of sensitive compounds—particularly high molecular weight organic and polymeric materials—the cryogenic approach employed by the matrix-assisted pulsed laser evaporation (MAPLE) technique enables their gentle transfer without structural damage. In this process, a rotating frozen target was ablated by a pulsed KrF* excimer laser (λ = 248 nm, pulse duration ≤ 25 ns) within a high-vacuum environment, facilitating the deposition of delicate materials onto substrates with minimal thermal impact.

The MAPLE experiments utilized a COMPexPro 205F KrF* excimer laser (Lambda Physics-Coherent), operating at a 15 Hz repetition rate. Initial tests explored various parameters—such as laser fluence, pulse frequency, substrate–target distance, and total number of laser pulses—to determine optimal deposition conditions. Following optimization, laser pulses with an energy of 360 mJ were directed onto a 27 mm^2^ spot using a beam homogenizer to ensure even energy distribution. The substrate and target were positioned in a parallel configuration with a separation of 5 cm. Each film deposition involved 100,000 pulses, and all experiments were carried out under a constant chamber pressure of 10^−4^ mbar in a stainless-steel vacuum system.

### 2.3. Thin Films Characterization Methods

#### 2.3.1. Scanning Electron Microscopy and Elemental Analysis

The morphology of the BG57+Mel thin films was investigated using a high-resolution Apreo S ThermoFisher (Hilsboro, CA, USA) scanning electron microscope (SEM), capable of achieving up to 0.7 nm resolution. For surface imaging, titanium substrates were employed, while Si wafers oriented along the (100) crystallographic plane were used to obtain cross-sectional views. Imaging was carried out at an accelerating voltage of 10 kV under a vacuum of 1 × 10^−3^ Pa. To reduce surface charging effects during observation, all samples were sputter-coated with a thin gold layer. Additionally, the same SEM system—equipped with a silicon-lithium (SiLi) energy-dispersive X-ray (EDX) detector—was used to assess the elemental composition of the deposited films.

#### 2.3.2. Atomic Force Microscopy

The surface topography of the BG57+Mel coatings was further characterized using atomic force microscopy in tapping mode. A Veeco atomic force microscopy (AFM) system was employed to scan a surface area of 5 µm^2^, with a resolution of 512 pixels and a scanning rate of 0.3 Hz. Measurements were performed using an RTESPA (Bruker, CA, USA) cantilever tip to accurately capture nanoscale surface features.

#### 2.3.3. Contact Angle Measurements and Surface Wettability

The hydrophilic or hydrophobic behavior of the MAPLE-deposited coatings was examined using the sessile drop technique. An optical tensiometer (Attension Theta Lite 101, Biolin Scientific, Sweden, software version 1.0.3) was used to measure static contact angles (CA) under ambient laboratory conditions (22 ± 1 °C and 42% relative humidity). The contact angle between simulated body fluid (SBF) droplets and the coating surfaces was determined to assess surface wettability. Additionally, roughness data were recorded during the measurements to support surface characterization.

#### 2.3.4. Electrochemical Behavior of the Samples

The electrochemical behavior of the samples immersed in simulated body fluid (SBF) test solution (at 37 °C) was evaluated by electrochemical impedance spectroscopy (EIS), using a VersaSTAT 3 potentiostat (Princeton Applied Research, Oak Ridge, TN, USA). In this experiment, data were recorded after 1, 12, and 24 h of immersion, respectively, by applying a sinusoidal signal of 10 mV RMS at open circuit potential, in a frequency range of 0.1 ÷ 1000 Hz. To perform the tests, a typical corrosion double-wall cell was used that consisted of a saturated Ag/AgCl electrode (as the reference electrode) (0.197 V), a platinum electrode (as the recording electrode), and the working electrode that consisted of the experimental samples (Ti used as reference and BG57+Mel). Data recording was performed using VersaStudio software (version 2.60.6, Princeton Applied Research, Oak Ridge, TN, USA), while in order to extract specific electrochemical parameters, EIS data were fitted using Zview software (version 12136-4, Scribner Associates Inc., Southern Pines, NC, USA). The EIS data were presented in the current study as Nyquist plots (imaginary vs. real impedance), Bode amplitude plots (lZl vs. frequency), and Bode phase plots (phase angle vs. frequency).

#### 2.3.5. Fourier Transform Infrared Spectroscopy

We used Fourier transform infrared (FTIR) spectroscopy to evaluate the chemical changes and surface transformations after different periods of immersion in SBF. Measurements were performed using the Shimadzu IRTracer 100 spectrometer in absorbance mode, operating in the 8000–400 cm^−1^ range with a resolution of 4 cm^−1^. Spectra were collected using the attenuated total reflectance (ATR) module. A total of 92 individual scans were recorded for each sample.

## 3. Results

### 3.1. Surface Investigation of As-Deposited Thin Films

**Figure 1:**
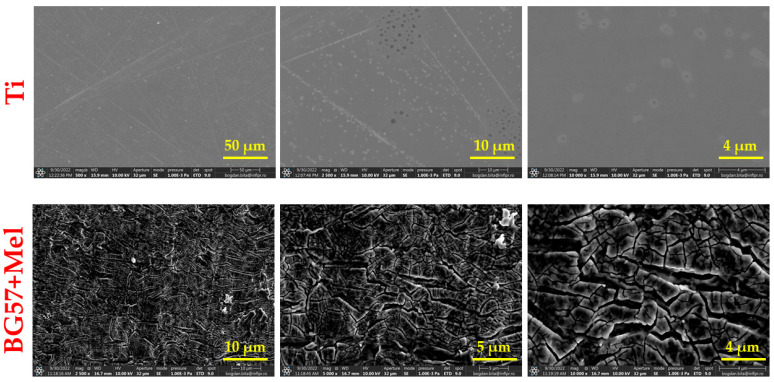
SEM micrographs of bare Ti substrates (first row) and BG+Mel MAPLE thin films deposited on Ti substrates (second row) in top view, at various magnifications.

Figure 1 displays SEM images illustrating the surface morphologies of BG57+Mel thin films, offering a comparative view across multiple magnifications.

Table 1 displays the 2D and 3D surface profiles of BG57+Mel samples, along with their corresponding roughness parameters.

The films exhibit uneven surface textures, with root mean square roughness (R_rms_) values of ~78.4 nm. These roughness measurements align with the SEM observations.

**Figure 2:**
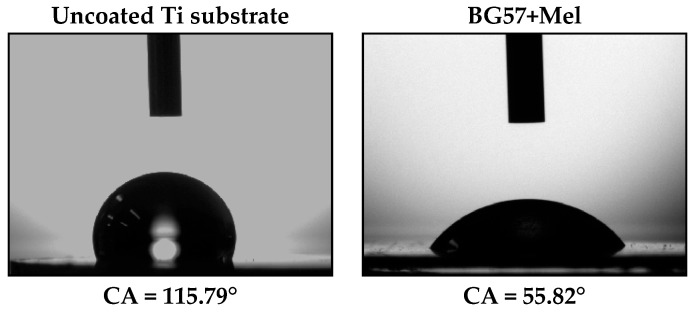
Surface wetting properties of the investigated samples.

The wettability of the obtained thin films was evaluated by measuring the CA between the sample surfaces (uncoated Ti and BG57+Mel) and SBF, as shown in Figure 2.

The bare Ti sample has a contact angle of 115.79° compared with 55.82° for Ti coated with BG57+Mel.

### 3.2. Electrochemical Performance of the Tested Samples

**Figure 3:**
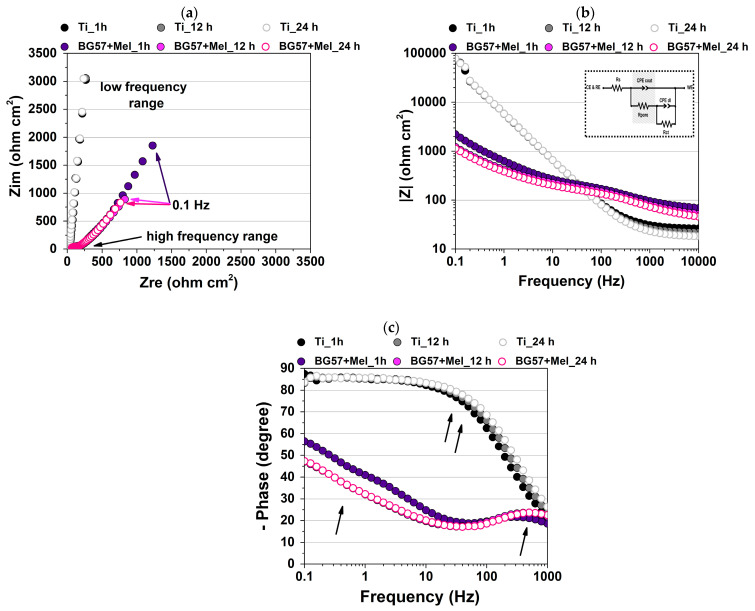
Nyquist (**a**), Bode amplitude (**b**) and phase plots (**c**) for Ti and BG57+Mel after 1, 12, and 24 h of immersion in SBF at 37 °C (additionally, the electrical equivalent circuit used for impedance data fitting is presented).

Figure 3 shows the impedance curves obtained, presented as Nyquist (a) and Bode amplitude/phase diagrams (b and c, respectively) for Ti and BG57+Mel as a function of SBF immersion time (i.e., 1, 12, and 24 h).

To have a detailed overview of the behavior of the investigated samples over immersion time, Table 2 presents data related to the increase/decrease in impedance data recorded at 0.1 Hz as compared with the initial spectra.

The extracted EIS fitted parameters are presented in Table 3.

### 3.3. Surface Investigation of Thin Films After SBF Immersion

We used FTIR spectroscopy to evaluate the chemical changes and surface transformations after 48 and 72 h, and after 5 and 7 days of immersion in SBF.

**Figure 4:**
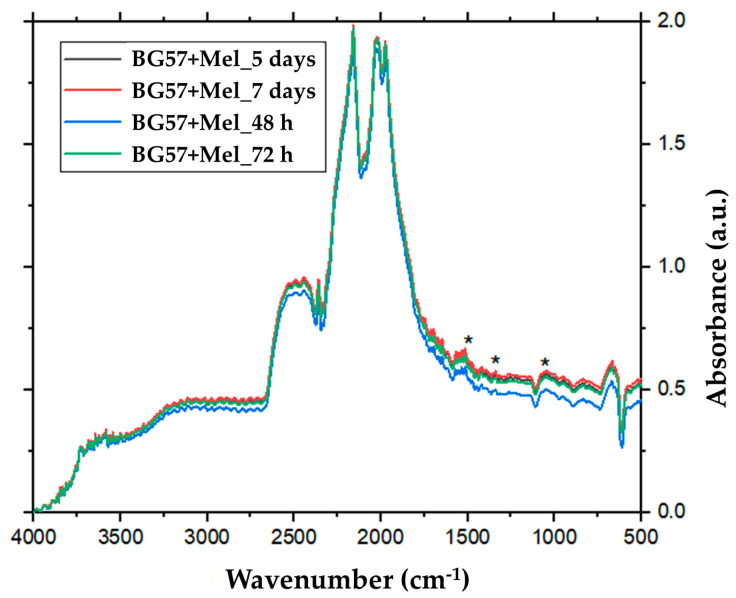
FTIR spectra of the samples after different immersion times.* represent the mail peaks of used substances.

After various immersion times in SBF, the surface transformations of the BG57+Mel samples were monitored. Surface morphology was examined using SEM. Images were captured at different magnifications on Ti substrates. The SEM images corresponding to the thin films are shown in Figure 4.

**Figure 5:**
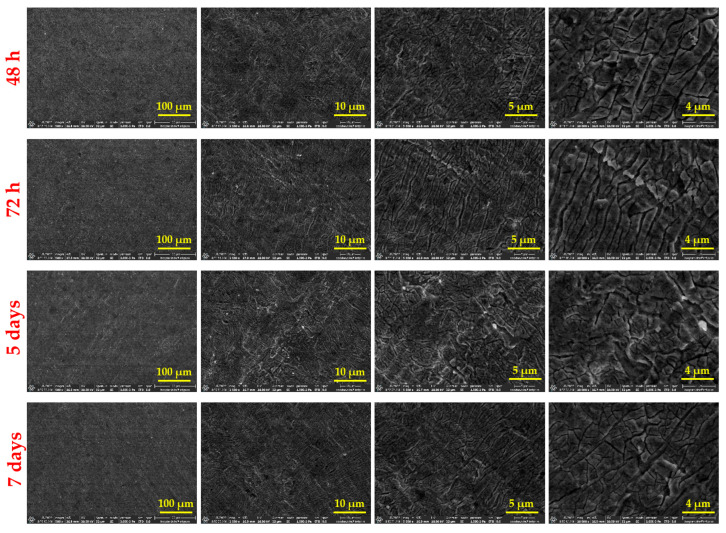
SEM images of BG57+Mel films after various immersion times.

After various immersion times in SBF, the surface transformations of the BG57+Mel samples were monitored using SEM analysis. Images were captured at various magnifications on coated Ti substrates. The SEM micrographs corresponding to the thin films are presented in Figure 5.

**Figure 6:**
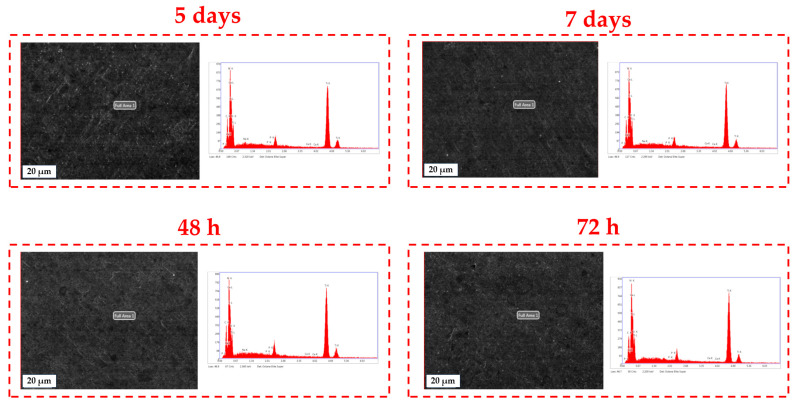
SEM-EDS analysis of BG57+Mel samples after various immersion times (scale 20 µm)/chemical element mapping in the selected microzone of interest.

The EDS analysis of BG57+Mel films after various immersion times was carried out using the backscattered electron TLD detector attached to the Apreo S ThermoFisher scanning electron microscope. In Figure 6, SEM images of the analyzed areas for the BG57+Mel samples are presented, along with the characteristic elemental spectrum from the selected microzone of interest.

In Table 4, we collected the EDS-derived elemental composition data of BG57+Mel coatings after various immersion times in SBF, highlighting the evolution of key elements involved in the mineralization process.

## 4. Discussion

The increasing incidence of implant poor osseointegration remains a critical challenge in the field of biomedical implants, necessitating the development of multifunctional coatings that can promote bone integration. In this study, BG57 and Mel coatings were successfully synthesized on titanium substrates using the MAPLE technique, resulting in uniform, chemically stable thin films that demonstrated structural integrity, favorable electrochemical behavior, and in vitro bioactivity as evidenced by the formation of an apatite-like layer in SBF.

### 4.1. Surface Investigation of As-Deposited Thin Films

SEM images at different magnifications (Figure 1) show that the BG57+Mel thin films have a dense, irregular surface morphology. Such micro-topography is advantageous for bone cell adhesion, making the material suitable for bone repair applications [35,36,37]. This micro-topography is known to favor osteoblast adhesion and proliferation, making such coatings promising for bone repair applications [38]. Similar findings have been reported by Jain et al., who observed that rough BG surfaces enhanced early osteoblast attachment and spreading compared to smooth surfaces [39]. This effect is attributed to increased surface area and the ability of surface irregularities to provide anchoring points for cellular filopodia, thereby facilitating stronger adhesion and mechanotransduction. Additionally, these features enhance the osteoconductive properties by supporting initial cell attachment and mineral deposition, aiding new bone formation around implants [40,41].

Textured or rough surfaces are known to promote cell adhesion, proliferation, and differentiation. These represent key processes in bone tissue regeneration [35,42,43]. Osteoblasts, essential for bone repair, adhere more effectively to surfaces with micro- and nano-scale features, which mimic the natural extracellular matrix. Such surface textures improve implant integration with surrounding tissue, especially in orthopedic devices like hip or knee replacements. Additionally, rough surfaces enhance protein adsorption from the biological environment, supporting stronger cell–surface interactions and signaling [44,45,46,47,48]. Gough et al. demonstrated that rougher 45S5 BG surfaces led to greater mineralized nodule formation and faster hydroxyapatite deposition in vitro, supporting the osteoconductive role of such textures [49]. These results align with our observation that, despite the surface irregularities, the films achieved uniform coverage without defects, maintaining morphological integrity.

Interestingly, while roughness promotes osteoblast differentiation and mineralization, some studies have reported a slight reduction in proliferation rate on highly rough surfaces [38], suggesting a shift towards a more mature osteoblastic phenotype, which represents a desirable outcome for implant integration. Therefore, the surface features of the BG57+Mel films may contribute not only to initial adhesion but also to downstream osteogenic events, reinforcing their potential in orthopedic applications. As expected, SEM micrographs of the uncoated Ti showed a smooth surface with minimal microstructural features, which further provides a baseline to evaluate the modifications introduced by the coatings. Further AFM micro-topography of BG57+Mel (Table 1) showed randomly distributed glass particles, specific to the intrinsic properties of the BG matrix, well known to enhance surface area and to promote osteoblast attachment, as previously mentioned. By comparing AFM results of BG57+Mel presented in the current study with those of a BG57 film (detailed topographical study published in a previous work [18], we can observe that both roughness parameters (Ra and Rrms) are lower for BG57+Mel. This result suggests that melittin incorporation leads to a smoother surface to some extent, reducing sharp height variation. Even if BG57 alone provides a textured surface favorable for osteointegration, the incorporation of Mel slightly alters the micro/nano-architecture, falling within the range considered optimal for osteoblast adhesion and differentiation. By comparing both groups, it is evident that Mel does not merely act as a bioactive agent but also contributes to textural refinement when co-deposited with BG57. This dual role—both chemical and physical—highlights the multifunctionality of the BG57+Mel system in optimizing implant surfaces for bone regeneration.

The substantial decrease in contact angle from 115.79° on bare Ti to 55.82° after coating with BG57+Mel (Figure 2) signifies a shift from hydrophobic to moderately hydrophilic surface behavior. This transition is driven by synergistic changes in both surface chemistry and topography, which enhance the material’s interaction with aqueous biological environments. The BG57+Mel coating introduces various hydrophilic functional groups to the surface, including hydroxyl (–OH), phosphate (PO_4_^3−^), and carboxyl (–COOH) groups from both the glass and the peptide. These polar groups enhance the surface free energy and increase the attraction between water molecules and the surface, promoting wetting behavior. Mel, being amphipathic, contributes additional hydrophilic domains that facilitate water spreading [50,51,52]. Such alterations have been shown to improve biocompatibility and cellular responses [53]. According to Wenzel’s equation, the presence of surface micro- and nano-topography amplifies the intrinsic wetting behavior of a material [54,55,56]. When a hydrophilic coating is applied to a rough surface, the effective contact area between water and the surface increases, further decreasing the contact angle [57]. This principle is supported by studies showing that multiscale roughened Ti surfaces with hydroxyapatite or oxide coatings significantly reduce contact angles and promote cell adhesion [58]. BG57 inherently forms silanol (Si–OH) groups upon contact with aqueous solutions, which further increase hydrophilicity. These groups attract and hydrogen-bond with water, enabling enhanced surface wetting and facilitating protein adsorption, which are critical for initial cell attachment and signaling in osseointegration [59]. Hydrophilic surfaces, in particular, tend to improve cell adhesion and support faster cell spreading [60,61]. In our case, the moderate wettability of the coating is favorable for osteoblast attachment and subsequent tissue integration, aligning with findings by Al-Noaman and Rawlinson, who reported that BG/graphene oxide composite coatings with contact angles below 20° exhibited enhanced cell adhesion and bioactivity on PEEK substrates [62]. Additionally, Maciąg et al. demonstrated that increasing the content of BG particles in polymer coatings modulates wettability and influences corrosion resistance, evidencing the interplay between BG content and surface energy [63]. Our results fit within this framework, as the BG57+Mel films maintained hydrophilic characteristics, which are essential for bioactivity and implant integration. Importantly, Spriano et al. also noted that bioactive glass coatings with contact angles around 80° still supported adequate cell viability and osteogenic potential, reinforcing that moderately hydrophilic surfaces like ours remain suitable for biomedical applications [64].

Taken together, these comparative insights confirm that the wettability of the BG57+Mel coatings is within the optimal range for promoting favorable cellular responses, validating the coating’s potential for orthopedic and dental implant applications.

### 4.2. Electrochemical Performance of the Tested Samples

Although in the Nyquist diagram a single semicircle is observed for all the investigated samples, which would suggest one time constant, the phase Bode diagram proves the presence of two phenomena that appear in different frequency areas (indicated by arrows). While two well-separated time constants were visible for BG57+Mel in both the high (~100 Hz, characteristic of a coating–SBF interface) and low frequency range (1–10 Hz, indicative of a substrate–SBF interface), Ti shows a broader peak in the middle region. Since previous studies reported the formation of an oxide layer on Ti immersed surfaces [65], it is reasonable to consider that in the current experiment, there is an overlap of the two phases. For example, Turdean et al. demonstrated that BG glass–chitosan composite coatings showed distinct impedance responses over time, with initial ion exchange at the coating surface followed by deeper substrate interactions, reflecting similar dual-phase behavior [66]. In the case of BG57+Mel, no significant phase shift related to the low frequency response was observed; only the peak present at high frequency becomes more noticeable over time, indicating ion exchange and higher bioactivity. Even though Ti exhibits higher charge transfer resistance and higher magnitude of impedance in the Bode plot (consistent with the Nyquist plot), the faster electrochemical reactions manifested by BG57+Mel and the fact that impedance slowly decreases over time can represent a beneficial electrochemical behavior in this case, targeting bone repair in tissue engineering applications.

Based on calculated values, after 12 h of immersion in SBF, the magnitude of the impedance data (lZl) showed a maximum decrease of 45% for BG57+Mel and only 4% for Ti at the lowest excitation frequency (0.1 Hz). This result may be due to the more pronounced degradation process exhibited by BG57+Mel in the first immersion period (i.e., 12 h), while Ti shows a certain stability, ascribed to a more passive oxide layer formation. This aligns with findings by Zurita-Méndez et al., who reported that BG–polymer scaffolds demonstrated enhanced electrochemical activity and progressive impedance reduction over time, corresponding to apatite formation and coating degradation in SBF [67]. Similarly, Edathazhe and Shashikala showed that phosphate glass coatings exhibited gradual impedance decreases during immersion due to electrolyte penetration and subsequent apatite development, paralleling the two-stage process described [68]. After this point, while Ti becomes more resistive, BG57+Mel shows no visible changes (only to a small extent as shown by the recorded lZl), reaching only a 49% decrease after 24 h, which indicates potentially stable performance in physiological conditions. The decreasing tendency over time can be explained by a two-stage effect: a) the penetration of the electrolyte through pores (inter-columnar voids) in the initial stage, which can initiate mass transfer reactions; followed by b) the blocking effect of the newly formed compounds [69].

For a quantitative interpretation, the recorded EIS data were fitted with an electrical equivalent circuit (EEC), presented as an inset in Figure 3b. The electrical components taken into consideration for the impedance data analysis were as follows: Rs = the solution resistance, CPEcoat = coating capacitance, Rcoat = resistance of the porous layer/coating, CPEdl = double layer capacitance, and Rct = charge transfer resistance. The above-mentioned circuit was selected based on physical significance and observed characteristics of the recorded data (i.e., the Nyquist plot showed depressed semicircles and time constants presented in the Bode phase plot). Since the ideal capacitor was replaced in the current study with a constant phase element (CPE), due to possible surface inhomogeneities [70], the impedance described by this parameter is defined by

$Z_{CPE}=1/{\left(j\omega \right)}^{\alpha }Q$ (ω = angular frequency, α = dimensionless parameter between 0 and 1, and Q  = non-ideal capacitance).

The low values of the χ2 parameter (i.e., ~10^4^) indicate a good agreement between the recorded impedance data and those simulated by the equivalent electrical circuit, indicating a reliable fitting procedure. As expected, due to SBF ingress, the Rs parameter exhibited a slight decrease over 24 h immersion in both cases, with higher values for BG57+Mel, possibly due to initial surface modification. The electrochemical parameters, which are indicative of the quality of the protection layers/coatings, are further highlighted (i.e., Qcoat + associated αcoat and Rpore). BG57+Mel showed a relatively high pore resistance (Rpore = 132–122 Ω cm^2^), indicating an improved barrier to ion transport, detrimental to the relatively low values of the oxide layer formed on the Ti surface (Rpore = 87–70 Ω cm^2^). With increasing immersion time, a decrease in this parameter is observed, allowing over time pathways formation towards the substrate, where a corrosion process could be initiated. While Ti shows a more capacitive character (~20 μF s^(α−1)^ cm^−2^) (with near-ideal stable αvalues—0.96), the Qcoat_BG57+Mel increased while associated αcoat decreased, suggesting the coating thickening over time (i.e., bioactivity).

The equivalent circuit fitting, with parameters such as Rpore and CPE behavior, provides key insight into the barrier properties of the coatings. As noted by Meng et al., a slight impedance reduction over time can signal beneficial bioactivity rather than coating failure, as bioactive surfaces encourage controlled ion exchange and biomineralization [71].

Relatively stable Qdl_Ti can be observed (Qdl~ 0.95 μF s^(α−1)^ cm^−2^, stable αdl = 0.93), while an increasing tendency of Qdl_BG57+Mel may suggest enhanced electrochemical activity at the interface with the substrate. The low values of αdl_ BG57+Mel (~0.6) suggest a deviation from ideal behavior and are consistent with the previous results. As previously mentioned, a more electrochemically active surface may represent a benefit in the current study, since BG57 coating with Mel addition presents a high potential of bioactive surface development, which is considered ideal for enhanced tissue interactions. The decreasing α values and increasing Qcoat for BG57+Mel, suggesting coating thickening and active surface behavior, are also well documented. Turdean et al. and Randviir and Banks et al. described how surface roughness and inhomogeneity (captured by CPE parameters) evolve with immersion time, indicating ongoing bioactivity and a transition from ideal to more complex electrochemical behavior [66,72].

To further highlight the specific impact of Mel in the present study, previous EIS data on BG57 were also considered [18]. The complete dataset provides a consistent baseline that allows direct comparison with the BG57+Mel system developed in the current study. As observed, the BG57+Mel coating exhibits higher Rs values than both Ti and BG57, regardless of immersion time. This suggests a denser film that restricts ionic diffusion into the electrolyte, reflecting enhanced protective capacity. While BG57 Ref shows much higher capacitance, indicating a more porous or defect-prone surface, the addition of melittin promotes a more compact and stable structure that reduces water uptake. The previous conclusion is also supported by αcoat and Rpore, which indicates improved homogeneity over BG57 alone, as well as suggesting that melittin controls pore integrity over time, maintaining, meanwhile, a bioactive surface complexity. Regarding Qdl and the associated electrochemical constant (i.e., αdl) of BG57+Mel, we can highlight a better-controlled electrolyte interaction at the interface, Mel effectively reducing the high capacitance of BG. To summarize, the EIS comparison confirms that melittin addition enhances the protective characteristics, improves film compactness, and maintains long-term performance, outperforming both Ti and BG57. These findings align with melittin’s known ability to modulate surface properties while stabilizing the complex electrochemical behavior of biomaterials used.

These results confirm that the BG57+Mel coatings not only serve as a physical barrier but also likely facilitate the development of a bioactive surface over time, a critical factor in ensuring long-term implant stability and osseointegration. Moreover, the similarity of our findings to previous studies on composite coatings highlights the effectiveness of the MAPLE deposition technique in producing uniform, adherent, and electrochemically stable bioactive films suitable for biomedical applications.

### 4.3. Surface Investigation of Thin Films After SBF Immersion

The FTIR spectra of the BG57+Mel coatings revealed characteristic absorption bands corresponding to both the BG matrix and the Mel, confirming successful incorporation of both components. Surface transformations of the BG57+Mel thin films were observed after 72 h of immersion in SBF (Figure 4), indicating the onset of BG dissolution and the formation of a carbonated apatite layer on the film surface. Characteristic peaks of carbonated apatite were detected at approximately 1515, 1335, and 1045 cm^−1^. The band at 1515 cm^−1^ was attributed to carbonate group absorption. The peak near 1335 cm^−1^ was assigned to phosphate absorption, while the 1045 cm^−1^ band corresponds to the asymmetric stretching of the P–O bond in (PO_4_)^3−^. This behavior is consistent with the apatite formation mechanism described in Ref. [73]. The amide I and II bands (~1650 cm^−1^ and ~1550 cm^−1^) evidenced the retention of Mel’s peptide backbone. These findings align with previous reports. For example, Spriano et al. demonstrated that chitosan-coated BG exhibited well-defined functional groups, and the coatings maintained their chemical signatures after immersion, validating FTIR as a robust tool for assessing coating stability [64]. Similarly, Civan and Nurbas identified phosphate and silicate phases in BG-alginate coatings, confirming that the appearance of characteristic vibrational modes indicates the material’s bioactivity potential [74]. Moreover, Mattos et al. highlighted the monitoring of apatite formation in mesoporous glass scaffolds [75]. The preservation of the amide bands in our spectra suggests that the MAPLE process did not degrade the Mel structure. This observation is consistent with Zarghami et al., who reported that Mel coatings on composite films retained functional group integrity, supporting their multifunctional bioactivity [15].

Taking into account the above-mentioned results, these comparisons reinforce that our results not only confirm the chemical composition of the BG57+Mel coatings but also support their expected biofunctional performance, validating the synthesis process and material stability observed in this study.

In the case of the SEM studies, 48 and 72 h intervals were selected to observe the initial nucleation and early formation of apatite-like mineral layers, which are expected to develop within the first 2–3 days for bioactive coatings in SBF. This timeframe was chosen to capture the progressive evolution of the coating’s surface morphology and its interaction with the surrounding environment, relevant to early implant integration and bioactivity assessment. Supporting this, Andersson et al. reported that calcium phosphate precipitation begins within 72 h on BG immersed in SBF, indicating significant mineralization can occur in this early window [76]. Similarly, Kawai et al. found that apatite precipitation on BG-coated Ti occurred within 2 days (48 h) in SBF, confirming rapid early mineralization in this period [77]. Moreover, Hamagami et al. observed the formation of initial apatite islands as early as 1 h after immersion, demonstrating that nucleation starts very early and continues progressively over the first 2–3 days [78]. These findings collectively support the selection of these early time points as critical intervals for evaluating the initial bioactivity of the coatings.

Immersion periods of 5 and 7 days were included to monitor the growth and maturation of the mineral layer, allowing us to evaluate whether the coating supports continued apatite formation over a longer period and to assess the stability and evolution of the film’s surface at biologically relevant timescales associated with early healing. This selection was based on studies showing that apatite maturation and crystallization occur progressively within this timeframe. Vallet-Regí et al. demonstrated that after 7 days of immersion in SBF, bioactive glass surfaces developed a well-formed apatite layer composed of needle-like crystallites, indicating significant maturation of the mineral layer by this time [79]. Similarly, Tirri et al. reported that by 5 to 7 days, bioactive glass-containing membranes became fully covered with a uniform calcium phosphate layer that transitioned from an amorphous phase to crystalline apatite, confirming the progression and maturation of the mineral phase [80]. Additionally, Thian et al. observed that nanostructured hydroxyapatite coatings developed a dense, mature apatite layer after 7 days of immersion in SBF, with structural and compositional characteristics similar to natural bone mineral [81]. These findings collectively support the use of these time points for assessing the ongoing bioactivity and surface evolution of the coatings under physiological conditions.

On the surface of the analyzed samples, regular formations can be observed, attributed to salt residues remaining on the thin film surface after immersion in SBF (Figure 5). Regardless of the immersion time, the surface morphology, characterized by a dense matrix with numerous irregularities, does not show significant visible changes. These salt deposits are likely the result of ionic interactions and precipitation processes occurring during immersion, particularly due to the supersaturation of calcium and phosphate ions in SBF, which can form apatite-like structures. The persistence of the irregular but stable matrix indicates good structural integrity of the coating throughout the immersion period.

The EDS spectra revealed peaks corresponding to Ti, the main element of the substrate. All recorded spectra show prominent peaks associated with the K and L lines of calcium (Ca) and phosphorus (P), confirming the presence of BG on the substrate. Changes in the atomic percentages of elements specific to BG were monitored (Table 3). The elemental analysis of BG57+Mel thin films after immersion in SBF revealed a progressive surface transformation indicative of bioactive behavior. In particular, the atomic concentrations of Ca and P increased notably during the first 48 h, which is consistent with the initial formation of a Ca–P layer, which is a precursor to carbonated hydroxyapatite (CHA). Such transformations are characteristic of bioactive glass dissolution followed by mineralization; a mechanism well-documented in the literature [82]. Specifically, Ca increased from 0.22% at 24 h to 0.43% at 48 h, while P peaked at 0.33% within the same time frame, suggesting active nucleation of phosphate-rich phases. Beyond 48 h, data from 5 and 7 days of immersion showed fluctuations in Ca and P content, with P decreasing to 0.13% at day 5 before rising again to 0.26% by day 7. Similarly, Ca levels showed a minor dip followed by recovery. These variations point toward dynamic processes of dissolution and reprecipitation, where initial calcium phosphate phases may dissolve and re-form as more stable apatite layers over time, aligning with previous findings on bioactive materials in aqueous environments. The presence of sodium (Na) increased slightly over time, which is indicative of the deposition of Na-containing salts from the SBF and is often observed during apatite formation in vitro [83]. Oxygen and nitrogen levels remained relatively stable, with minor fluctuations in N likely due to contributions from the organic component Mel, known to contain nitrogen-rich residues. Carbon content also remained consistent, supporting its origin from both the organic matrix and carbonate species associated with the formation of carbonated apatite.

Taken together, the elemental evolution observed by EDS provides strong evidence for the bioactivity of BG57+Mel coatings. The increasing Ca and P content, combined with the emergence of apatite-like features, confirms their potential to support osteoconduction and bone bonding, which are crucial for biomedical implant applications.

Although the present study focuses on the combined BG57+Mel coating, the behavior of BG57 alone in SBF has been extensively studied in previous works. For instance, Floroian et.al demonstrated that BG57 coatings exhibited significant bioactivity, characterized by rapid apatite formation on substrates immersed in SBF. The findings confirmed the development of a continuous, bone-like mineral layer within just a few days of immersion, highlighting BG57’s inherent osteoconductive potential and its stability in physiological environments [84]. Additional studies have shown that BG57 coatings enhance corrosion resistance, making them suitable as standalone coatings for orthopedic implants [85].

The incorporation of Mel into BG57 coatings raises the question of whether its presence might influence the stoichiometry of the BG or alter the kinetics of apatite formation. Our EDX results confirmed the stable presence of Si, Ca, and P as expected for BG57, while FTIR spectra showed preserved Si–O–Si and P–O characteristic peaks, with additional amide bands indicating Mel’s successful incorporation. These observations suggest that the fundamental glass network remains intact despite the addition of Mel, in line with findings by Park and Lee, who demonstrated that Mel can be integrated into composite biomaterials without disrupting the primary matrix structure [86].

Furthermore, the progressive formation of apatite-like mineral layers observed during SBF immersion did not exhibit significant delays or abnormalities compared to pure bioactive glass systems. This is consistent with observations that Mel, while biologically active, primarily influences microbial membranes and does not chemically interfere with inorganic mineralization pathways [87]. Although some studies suggest Mel can interact with lipid bilayers and potentially impact local ionic environments [88], no measurable alteration in apatite kinetics was noted in our coating system within the tested timeframe.

Overall, these findings suggest that Mel’s integration enhances the coating’s functional bioactivity without compromising its chemical integrity or bioactive performance, supporting its potential as a multifunctional additive in BG coatings.

## 5. Conclusions

Based on the results obtained, the main conclusions of this study are as follows:

- MAPLE-deposited composite coatings composed of BG57 and Mel significantly enhance the surface properties and bioactivity of Ti substrates, making them promising candidates for biomedical implant applications.
- The resulting thin films exhibited a rough, nanoscale-textured morphology, which is favorable for supporting cell adhesion and tissue integration.
- Contact angle measurements confirmed a marked improvement in surface wettability, with the coatings transitioning from hydrophobic to moderately hydrophilic behavior.
- EIS demonstrated enhanced corrosion resistance in SBF, indicating the protective effect of the BG57+Mel coating.
- FTIR and EDS analyses confirmed the gradual formation of a carbonated apatite layer over time, supporting the material’s osteoconductive potential.
- The integration of Mel successfully functionalized the surface without compromising the structural integrity of the films, highlighting the benefit of MAPLE for depositing thermally sensitive organic–inorganic composites.
- The study’s limitations include the absence of direct antimicrobial and cytocompatibility testing, the exclusive use of SBF immersion to assess bioactivity, and testing under static in vitro conditions only. These limitations emphasize the need for future research, which should focus on cytocompatibility and cell viability assays, following established methodologies as outlined in systematic reviews such as Valenti et al., to fully validate the coatings’ biocompatibility [89].

In summary, the BG57+Mel coatings not only improved surface reactivity and corrosion resistance but also exhibited bioactive behavior essential for osseointegration. These findings support the future development of multifunctional coatings for orthopedic and dental Ti implants.

## Figures and Tables

**Table 1:** 2D and 3D AFM images of the sample surface recorded on 5 µm^2^ surfaces along with the roughness parameters.

Sample	2D Image	3D Image	Roughness Parameters (nm)	
			Ra	R_rms_
BG57+Mel	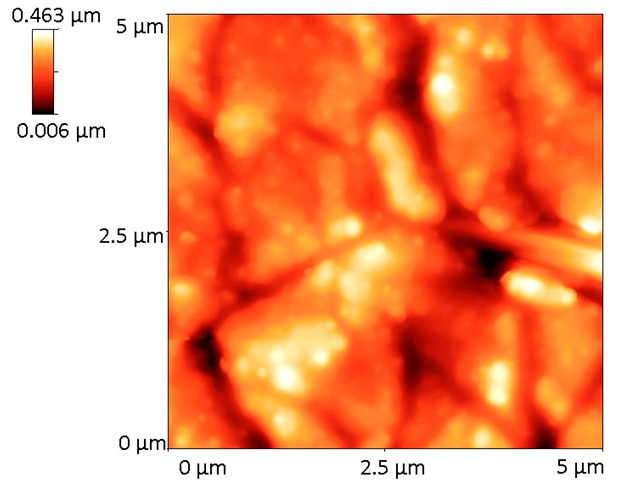	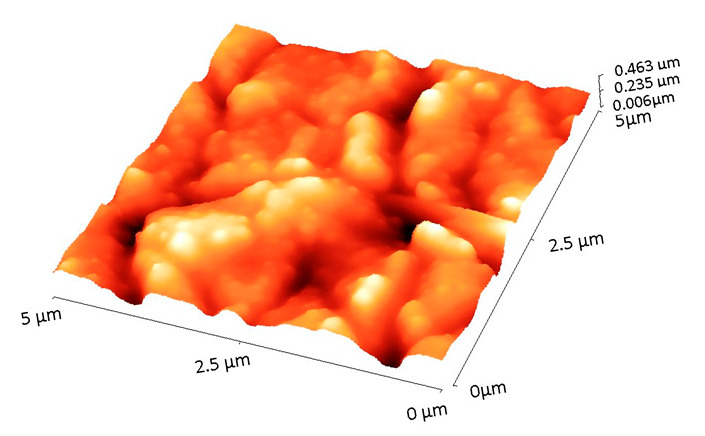	**62.0**	**78.4**

**Table 2:** Impedance data recorded at 0.1 Hz and the stability in time of the investigated samples.

Samples	lZl (Ω) @0.1 Hz	Stability in Time (% Decrease @0.1 Hz)
Ti_1h	75,163	-
Ti_12h	72,218	4
Ti_24h	78,868	-5
BG57+Mel_1h	2221	-
BG57+Mel_12h	1213	45
BG57+Mel_24h	1127	49

**Table 3:** EIS fitted electrochemical parameters of Ti and BG57+Mel after 1, 12, and 24 h of immersion in SBF at 37 °C.

Samples	Time	Ti	BG57+Mel
Rs (Ω cm^2^)	1 h	27	69
	12 h	23	47
	24 h	19	41
Qcoat (μF s^(α−1)^ cm^−2^)	1 h	19.89	28.636
	12 h	19.95	47.089
	24 h	20.25	49.223
αcoat	1 h	0.96	0.77
	12 h	0.96	0.70
	24 h	0.96	0.69
Rpore (Ω cm^2^)	1 h	87	132
	12 h	75	139
	24 h	70	122
Qdl (μF s^(α−1)^ cm^−2^)	1 h	9.90	623.16
	12 h	9.68	1199.70
	24 h	9.13	1300.70
αdl	1 h	0.93	0.60
	12 h	0.93	0.56
	24 h	0.93	0.56
Rct (Ω cm^2^)	1 h	-	-
	12 h	-	-
	24 h	-	-
χ^2^	1 h	2 × 10^−4^	3 × 10^−4^
	12 h	2 × 10^−4^	1 × 10^−4^
	24 h	2 × 10^−4^	1 × 10^−4^

**Table 4:** Atomic percentages of key elements in BG57+Mel films after different immersion times in SBF.

Immersion Time	Key Elements in BG57+Mel Films					
	%C	%N	%O	%Na	%P	%Ca
24 h	9.37	13.16	8.85	0.02	0.27	0.22
48 h	10.27	12.88	7.79	0.02	0.33	0.43
5 days	9.71	14.46	7.6	0.15	0.13	0.25
7 days	9.92	13.92	9.77	0.11	0.26	0.39

## Data Availability

The original contributions presented in the study are included in the article; further inquiries can be directed to the corresponding author.
